# Drink and Heredity

**Published:** 1899-07-29

**Authors:** 


### Drink and Heredity.

In opening a discussion the other day at the Society for the Study of Inebriety, Professor Sims Woodhead drew attention to the bearing of modern theories regarding heredity upon the drink question. The two hypotheses which he especially set himself to controvert were, first, that the taste for drink is transmitted from father to son—in other words, that children are born with such an innate tendency to drink that they are hardly to be held accountable when in later years they become drunkards; second, that a drinking nation gradually develops a sort of immunity to drink, so that, bad as may be the effects of alcohol on the present generation, the drunkenness of to-day does but lead to the temperance of to-morrow, and to the gradual development of a race which will not only be immune to the effects of alcohol, but indifferent to its charms. To our mind there is a dash of the academic about the whole discussion. Still, although we can hardly conceive of a man drinking for the sake of posterity, we can readily imagine one who knows his weakness excusing himself by throwing the blame upon his ancestors, and claiming, when he fails, that it is not his fault; and, seeing the eagerness with which people nowadays grasp at "scientific" excuses for all their little peccadilloes, Professor Woodhead has no doubt done well in showing how little excuse is provided by either of these hypotheses for intemperance in the use of alcohol. In regard to the question of immunity, Professor Woodhead drew a broad distinction between that resistance to the evil effects of alcohol on the tissues which, he thought, might possibly, at the end of many generations, be induced, and that indifference to its "taste," or that increased power to withstand its temptations, which some people believe is likely to be the outcome of such prolonged indulgence in it. He said that a man unaccustomed to take alcohol was, no doubt, much more readily affected by it physiologically than after he had taken it for a lengthened period. That was granted. His tissues were able to carry on their work more easily in the presence of alcohol after they had become accustomed to it than before, but that had nothing to do with the question of the taste for alcohol, or with the power of the future generations to withstand the temptations offered by it. Indeed, he held that, even so far as the acclimatisation of the tissues was concerned, that end was always attained, when it was attained, at the expense of the more highly organised tissues and their functions; and it seemed to him entirely unreasonable to believe that this sort of immunity, which after all was but a sort of compensation, a carrying on of a less perfect life under a heavier burden, could lead to a higher development of the highest powers, namely those of self-control. But, turning to another side of the question, he asked, supposing that by the continued use of alcohol a certain immunity to its effects could be obtained, and supposing, a thing which, however, he would not grant, that by the hereditary transmission of tendencies an immunity against drunkenness might be reached in times to come, was it worth while? Take even the extreme case of vaccination against smallpox. No one, he said, was more strongly in favour of vaccination than he was. But even in regard to vaccination there were certain inherent evils to be faced. The desirability of obtaining immunity from small-pox in such a manner hinged entirely upon the impossibility of obtaining it in other ways, and he thought no argument founded upon the immunity to small-pox given by vaccination was applicable in any way to a condition such as alcoholism, the evils of which we could escape by an exercise of will and self-control. This line of thought brought him back to the other hypothesis with which he desired to deal, namely, that the taste for drink is transmissible by heredity. This he entirely denied. He held most strongly that a direct transmission of the taste for alcohol never occurred. Of course he accepted very fully the fact that certain nervous diseases and degenerations involving certain altered and weakened inhibitory powers are transmitted from generation to generation. These, however, did not always assume the same form, the manifestation of the defect often taking on very different characters in different generations. But whatever character they assumed, the result, as regards alcohol, was inevitably the same; and until far more evidence was brought forward than had yet been presented, he should strongly maintain that, what was so often spoken of as an inherited taste for alcohol, was an inherited weakness and lessened self-restraint, affecting many other things besides drink, and that a direct transmission of the taste for alcohol from parents to children in a constitution otherwise healthy did not occur.

As with tuberculosis the disease was not transmitted, but only the weakly and unbalanced condition of the tissues. We do not say how far the views of Professor Sims Woodhead will meet with general acceptance. So far as concerns the impracticability, nay, the undesirability of driving away the taste for alcohol by inuring the tissues to its effects, we are entirely at one with him; but when it becomes a matter of measuring up the responsibility of the individual there seems but little to choose, so far as the "patient" is concerned, between an inherited taste for alcohol and an inherited weakness which makes it difficult to keep away from it.

